# Tumorigenesis by Meis1 overexpression is accompanied by a change of DNA target-sequence specificity which allows binding to the AP-1 element

**DOI:** 10.18632/oncotarget.4488

**Published:** 2015-08-03

**Authors:** Leila Dardaei, Dmitry Penkov, Lisa Mathiasen, Pranami Bora, Marco J. Morelli, Francesco Blasi

**Affiliations:** ^1^ IFOM, FIRC Institute of Molecular Oncology, IFOM-IEO Campus, Milano, Italy; ^2^ Department of Experimental Cardiology, Russian Cardiology Research and Production Complex, Moscow, Russia; ^3^ Center for Genomic Science of IIT@SEMM, Fondazione Istituto Italiano di Tecnologia (IIT), Milan, Italy; ^4^ Present Address: Massachusetts General Hospital Cancer Center, Harvard Medical School, Charlestown, MA, USA

**Keywords:** Meis1, Prep1, AP-1, tumorigenesis, ChIP-seq

## Abstract

Meis1 overexpression induces tumorigenicity but its activity is inhibited by Prep1 tumor suppressor. Why does overexpression of Meis1 cause cancer and how does Prep1 inhibit? Tumor profiling and ChIP-sequencing data in a genetically-defined set of cell lines show that: 1) The number of Meis1 and Prep1 DNA binding sites increases linearly with their concentration resulting in a strong increase of “extra” target genes. 2) At high concentration, Meis1 DNA target specificity changes such that the most enriched consensus becomes that of the AP-1 regulatory element, whereas the specific OCTA consensus is not enriched because diluted within the many extra binding sites. 3) Prep1 inhibits Meis1 tumorigenesis preventing the binding to many of the “extra” genes containing AP-1 sites. 4) The overexpression of Prep1, but not of Meis1, changes the functional genomic distribution of the binding sites, increasing seven fold the number of its “enhancer” and decreasing its “promoter” targets. 5) A specific Meis1 “oncogenic” and Prep1 “tumor suppressing” signature has been identified selecting from the pool of genes bound by each protein those whose expression was modified uniquely by the “tumor-inducing” Meis1 or tumor-inhibiting Prep1 overexpression. In both signatures, the enriched gene categories are the same and are involved in signal transduction. However, Meis1 targets stimulatory genes while Prep1 targets genes that inhibit the tumorigenic signaling pathways.

## INTRODUCTION

Changes of expression of oncogenes and tumor suppressors can cause cancer. However, it is not clear why the elevated levels of some transcription factors promote cancer. Although high throughput studies examining the oncogene or tumor suppressor activity of DNA binding proteins are available [1, 2], no study has addressed the effect of the expression level on the DNA binding selectivity.

Myeloid ecotropic insertion site 1 (Meis1) is a DNA binding transcription factor of the TALE (three amino acid loop extension) homeodomain family [3, 4], a potent oncogene in leukemia and solid cancer [5]. In contrast to oncogenic Meis1, its closely related TALE family member, Pbx-regulating protein 1 (Prep1, aka pKnox1), is a tumor suppressor in mice and humans [6, 7]. Prep1 basic tumor suppressive mechanism is the maintainance of the genomic stability [8], a very important hallmark of tumorigenesis [9]. Meis1 and Prep1 compete for pre-B-cell leukemia homeobox 1 (Pbx1) determining the tumorigenic fate of mouse embryonic fibroblasts (MEFs). Prep1 reduces Meis1 transcriptional activity and inhibits tumorigenicity [10].

In this paper we have used a set of five isogenic MEF cell lines expressing different amounts of Meis1 and/or Prep1 to understand how the increased intracellular concentration of Meis1 or Prep1 leads to or inhibits cancer, respectively.

We show that the number of Meis1 and Prep1 binding sites directly correlates with their expression level, second, that overexpression of Meis1 increases seven fold the number of Prep1 enhancer targets and, third, drastically changes the consensus Meis1 DNA binding motif. Upon overexpression in MEFs, the enriched DNA sequence is that of the AP-1 element, a target of the Jun/Fos family of transcription factor. Finally by integrating ChIP-seq and RNA-seq data from the various cell lines we identify a tumor-specific Meis1 signature and a suppression-specific Prep1 signature. Gene Ontology analysis of these signatures uncovers that Meis1 and Prep1 target the same gene categories regulating transcription and signal transduction. However, while Meis1 target genes stimulate, Prep1 targets inhibit these processes.

## RESULTS

### The number of Meis1 and Prep1 DNA binding sites is proportional to their level of expression

**Figure 1:**
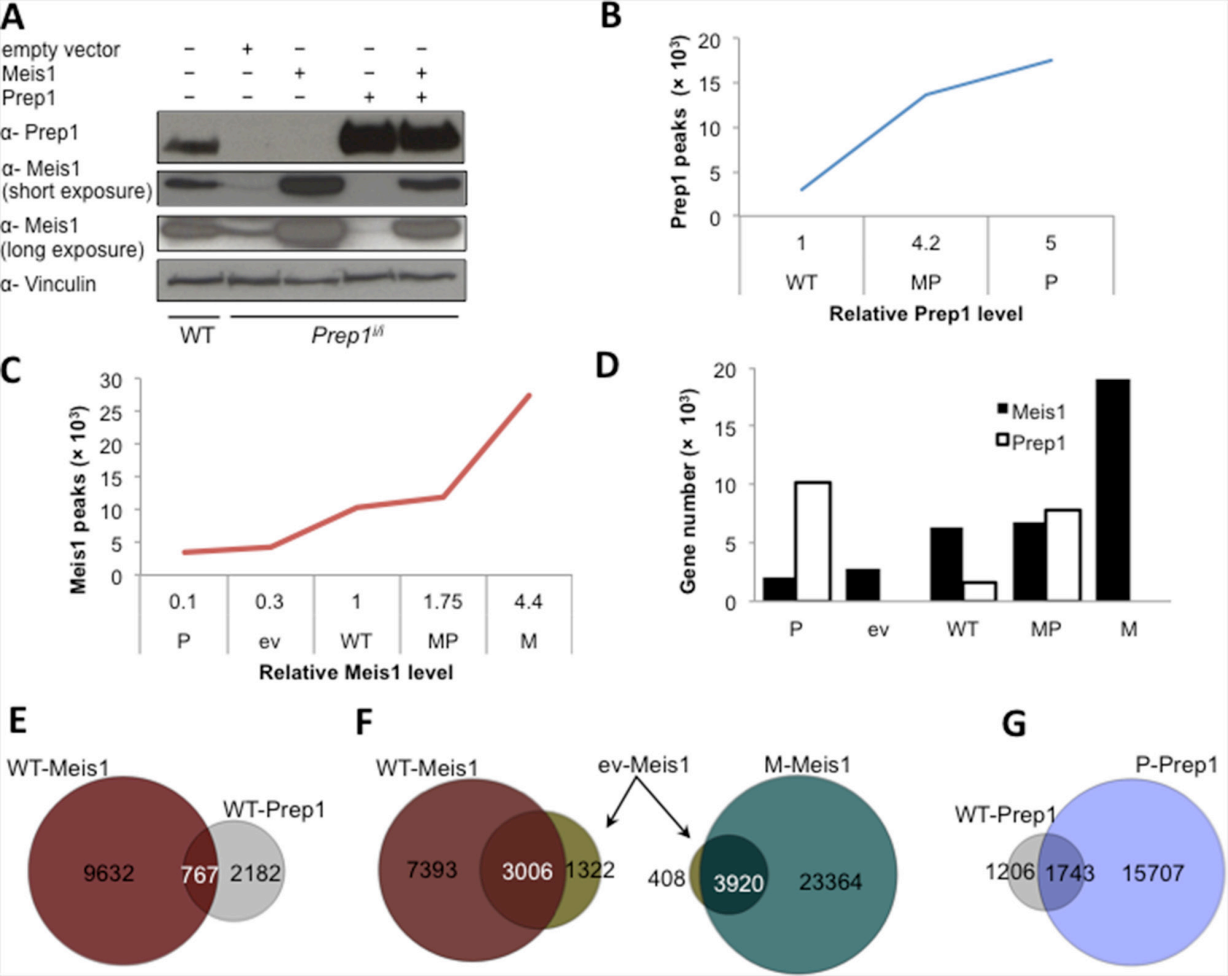
DNA binding profiles of Meis1 and Prep1 in MEFs **A.** Western blot determining the relative levels of Meis1 and Prep1 in five different cell-types. Vinculin was used as loading control. **B.** Concentration-dependent binding of Prep1. Number of significant Prep1 peaks in WT, MP and P cells is shown relative to its protein levels (*p* < 10^−6^). **C.** Concentration-dependent binding of Meis1. Number of significant Meis1 peaks in P, ev, WT, MP and M cells is shown relative to its protein levels (*p* < 10^−6^). **D.** Graph shows the number of Meis1 and Prep1 target genes in the five cell-types. Ev and M cells express no Prep1 (refer to panel A). **E.** Venn diagram showing the overlap between Meis1 and Prep1 peaks in WT cells. **F.** Venn diagrams showing overlap of Meis1 peaks in ev and WT (left) or ev and M (right) cells. **G.** Venn diagram showing overlap of Prep1 peaks in WT and P cells.

We performed ChIP-seq (chromatin immuno-precipitation coupled to DNA sequencing) with antibodies specific for Meis1 or Prep1 in five different isogenic cell lines bearing single gene differences. Hypomorphic *Prep1^i/i^* MEFs (7) were infected either with an empty vector (ev cells) or with *Meis1* (M cells) or *Prep1* (P cells) expression vectors. Only the M cells become tumorigenic [10]. The M cells, further infected with a *Prep1* expression vector, generated the MP line that still produces tumors *in vivo* but is strongly inhibited. Ev, M and MP cells have been described previously [10]. Littermate WT MEFs are a reference cell line for ChIP-seq and RNA-seq experiments. This set of five cell lines have different levels of Meis1 and Prep1. The level of expression of Meis1 and Prep1 in the various cell lines, relative to WT, was measured by immuno-blotting and is shown in Figure 1A. As previously described [10], Meis1 decreases both in the absence (ev and M cells) and upon overexpression of Prep1 (P cells).

ChIP-seq data (deposited in Gene Expression Omnibus (GEO), accession numbers GSE54221 and GSE58802) were filtered using as threshold of significance a *p*-value < 10^−6^ (based on the difference in reads between the immuno-precipitated and input samples). The total number of ChIP-seq peaks varied with the protein levels. In WT cells, Prep1 bound 2,949 genomic regions whereas in MP (expressing 4.2-fold more Prep1 than WT) and P cells (5-fold more Prep1) it bound 13,562 and 17,450 sites, respectively (Figure 1B). Prep1 binding sites were not measured in ev and M cells because of the absence of Prep1.

Likewise, in WT cells Meis1 bound to 10,399 sites but this number decreased in the Prep1-hypomorphic ev cells (4,328 peaks), in which Meis1 level is 30% of WT and even more (3473) in P cells (Meis1 level being 10% of WT) in which Prep1 is overexpressed (Figure 1C). In M cells, which express 4.4-fold more Meis1 than WT (and no Prep1), the number of Meis1 peaks increased 2.6-fold over WT (27,284 peaks). In MP cells, which express 1.75-fold more Meis1 than WT and overexpress Prep1, Meis1 bound to a much lower number of sites (12,008 peaks). As shown in Figure 1C, the number of Prep1 and Meis1 binding sites directly correlated with the level of the transcription factors. The same data are reported in Figure 1D in terms of target genes rather than peaks. Again, the number of target genes is directly related to the level of expression.

In conclusion, the number of peaks or of genes bound by Prep1 and Meis1 is clearly in direct correlation with the intracellular level of the protein.

### Prep1 and Meis1 at various expression levels occupy a small number of core target sites

DNA binding conditions in MEFs must be very different from those of the cells of the whole embryo trunk [11] and of embryonic stem cells [12] as the ChIP-seq analysis of Prep1 and Meis1 shows important differences. In WT MEFs, 26% of Prep1 peaks overlap with those of Meis1 and 7.3% of the total Meis1 peaks overlap with those of Prep1 (Figure 1E). Thus, a quarter of Prep1 and less than one tenth of Meis1 sites share the same regions. This result is only in partial agreement with the previously published data on the E11.5 embryo trunk [11], which recorded a lower overlap for Prep1 (12%) but a similar overlap for Meis1 (7.1%). Thus in MEFs Prep1 occupies a larger proportion of the Meis1 binding landscape.

In ev cells the number of Meis1 DNA-binding peaks (Figure 1C) is reduced, but over 70% of these peaks overlaps with those bound in WT (Figure 1F, left). In M cells, Meis1 overexpression increases the number of Meis1 peaks 6.3-fold over ev cells (Figure 1C), a number that includes 90% of the peaks bound in ev cells (Figure 1F, right). Likewise, 60% of Prep1 peaks in WT cells overlaps with those in Prep1-overexpressing P cells (Figure 1G). Therefore Meis1 and Prep1 bind to a core set of binding sites preserved under different expression conditions, but their binding repertoire is enlarged by overexpression.

### Overexpression of Prep1, not of Meis1, increases the relative frequency of binding to TSS-remote regions

We divide Meis1 and Prep1 peaks in transcription start site-associated (TSSA) if located within −500 to +100 bp from a TSS, intragenic (IG) if located within a transcription unit and intergenic if outside of a transcription unit. Close intergenic (CI) are < 20 Kb away, and far intergenic (FI) > 20 Kb from the TSS of an annotated gene. This subdivision may have functional relevance, because it tends to separate promoters from enhancers.

The distribution of Meis1 peaks in the four genomic locations is essentially the same in WT, ev and M cells (Supplementary Figure S1A): Meis1 binds preferentially to FI (about 55% in the three different cell types) IG (about 27%) and CI (18%) regions. A small percent, on average 0.89%, of Meis1 peaks, binds close to a TSS (Supplementary Figure S1A). Thus neither the absence of Prep1 (compare ev and WT cells) nor its own overexpression (compare ev and M cells) affect the genomic distribution of Meis1 binding sites i.e. the preferential binding to far intergenic regions.

In WT cells, Prep1 peaks in the TSSA group are more abundant than Meis1 (3.35% vs. 0.78%), but are less frequent in the CI group (7.66% vs. 16.36%) (Supplementary Figure S1A and S1B) and show similar distributions in FI and IG regions (Supplementary Figure S1B). Upon Prep1 overexpression (P cells) the percent of Prep1 peaks in the TSSA and FI group decreases while increasing in the CI group (Supplementary Figure S1B). Therefore, unlike Meis1, the distribution of Prep1 peaks changes with the Prep1 level.

The overall distribution pattern of Meis1 peaks agrees with the previously published data on total E11.5 embryo trunk [11] in which Meis1 peaks are mostly located in “TSS-remote regions”. On the contrary, the distribution of Prep1 peaks in WT MEFs is different from that in the E11.5 embryo trunk since only 3.35%, v. 30%, is located in the TSSA group. Prep1 peaks are more abundant in the FI regions raising from almost 18% in the E11.5 embryo [11] to 60% in MEFs. Therefore, although ubiquitously expressed, Prep1 displays a cell-type dependent occupancy of functional genomic regions. This may have great relevance in Prep1 transcriptional regulation since the TSSA-remote binding sites may well include functionally different, enhancer-like sequences.

Overall, the distance of the Meis1 and Prep1 peaks from the closest TSS, shows a similar distribution in the different cell types analyzed (Supplementary Figure S1C and S1D).

### Meis1 does not profoundly affect the binding landscape of overexpressed Prep1, whereas Prep1 prevents binding of overexpressed Meis1

**Figure 2:**
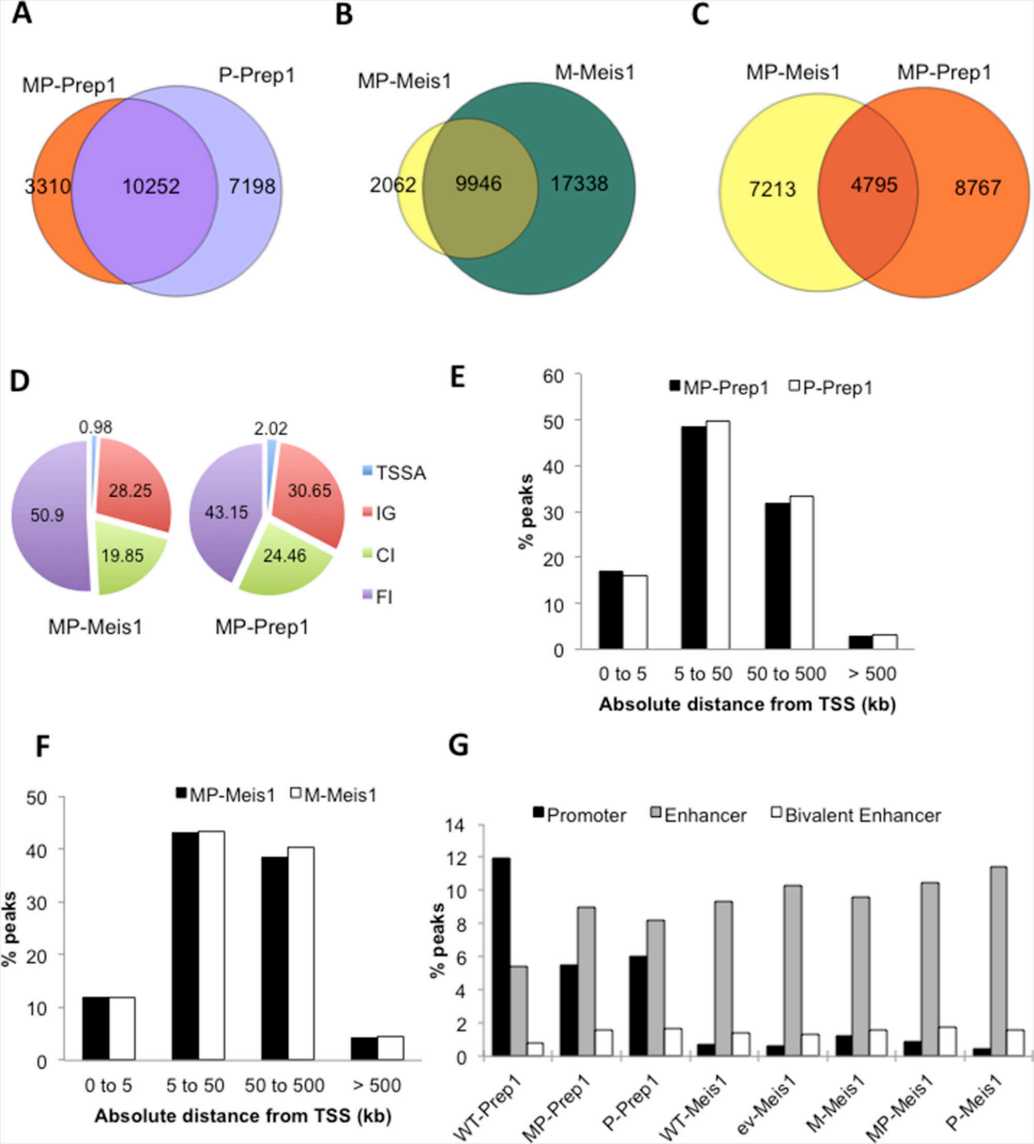
Overexpression modifies the enhancer preference of Prep1, not of Meis1 Venn diagrams showing the overlap of **A.** Prep1 peaks in MP and P cells, **B.** of Meis1 peaks in MP and M cells and **C.** of Meis1 and Prep1 peaks in MP cells. **D.** Pie chart showing that overexpressed Prep1 has twice as many TSS-associated peaks than Meis1 in MP cells. **E.** Overexpression of Prep1 in MP and P cells does not change the TSS-distance distribution of peaks. **F.** Overexpression of Meis1 in MP and M cells does not change the TSS-distance distribution of peaks. **G.** Prep1 overexpression, but not Meis1 overexpression changes the percent occupancy of regions containing promoter and enhancer marks in the various cell-types.

ChIP-seq shows 23% less Prep1 binding sites in MP cells, 13,562 v. 17,450, in agreement with the decrease of Prep1 (compare MP to P cells), with an extensive overlap (Figure 2A). Likewise, Meis1 peaks (compare MP to M cells) decrease 2.5-fold (27,284 in M versus 12,002 in MP, Figure 2B), comparable to the decrease in protein level (Figure 1A). However, the percent of Prep1-Meis1 overlapping peaks increases from 7.3% in WT (Figure 1E) to about 40% in MP cells (Figure 2C). Thus overexpression of Prep1 causes binding to many regions shared with Meis1.

### Prep1 and Meis1 levels modulate their promoter versus enhancer binding frequency

Prep1 TSSA-associated peaks in the MP cells was twice higher (2.02%) than for Meis1 (0.98%) (Figure 2D). Moreover, neither the single overexpression of Meis1 or Prep1 (M and P cells), nor their coexpression (MP cells), changed the distribution of the peaks in relation to their distance from the closest TSS (Figure 2E and 2F). Thus, Prep1 induces major changes in the number but not in the genomic distribution of Meis1 peaks in MP MEFs whereas overexpression of Meis1 poorly affects either the number or the genomic distribution of the Prep1 peaks. In E11.5 embryo trunks, 30% of Prep1 versus 3% of Meis1 peaks coincide with the presence of promoter marks (RNA Pol II and H3K4Me3^+^), whereas 26% of Meis1 peaks and 12% of Prep1 peaks overlap with the H3K4Me1^+^ enhancer mark [11]. In MEFs, the same analysis yielded different results. In WT MEFs, 12% of Prep1 and 0.7% of Meis1 peaks overlapped with promoter marks, whereas 5.4% of Prep1 and 9.3% of Meis1 peaks coincided with enhancer marks (Figure 2G). Thus the differential preference of Prep1 for promoters and of Meis1 for enhancers [11] was present also in MEFs but was less pronounced. When Prep1 was overexpressed, regardless of Meis1 level (MP v. P), the frequency of Prep1 promoter peaks was reduced by 50% while the enhancer peaks increased about seven-fold to 35%. Under these conditions, also bivalent enhancers (H3K4me1^+^; H3K27Ac^+^) increased 50% (Figure 2G). Despite the different level in ev, M, MP, P and WT cells, the relative frequency of Meis1 binding to enhancers (almost 10% of the peaks in all five cell types) and bivalent enhancers (1.5%) did not change. However, its enrichment at promoter regions slightly increased with the increase of its level (Figure 2G).

Therefore overexpression leads to more frequent binding of Prep1 to enhancer, and minor change of Meis1 binding to promoter sites.

### Overexpressed Meis1 binds an AP-1-specific DNA consensus

ChIP-seq on the whole embryo trunk and on embryonic stem cells was enriched in the binding consensus for Prep1 and Meis1: a decameric consensus sequence (TGAXTGACAG, DECA) for Prep1, or an octameric TGATXXAT (OCTA) and an hexameric TGACAX (Hexa) for Meis1 [11–13].

**Figure 3:**
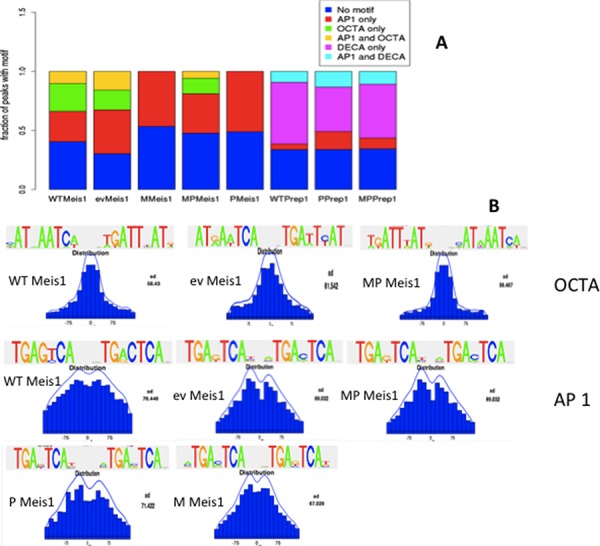
Meis1 and Prep1 binding sites in MEFs are enriched in AP-1 consensus DNA sequences **A.** rGADEM OCTA and AP-1 motifs frequency in Meis1 and Prep1 ChIP-seq in the five cell lines. **B.** Position of the OCTA and AP-1 motifs in the 300 bp-large peaks.

In our MEF lines Meis1 and Prep1 consensus sequences changed (see Methods in Supplementary section) when proteins were overexpressed. Prep1 DECA consensus was always most enriched in all cells; however, Meis1 binding consensus, in addition to OCTA included in all cell lines a typical heptameric AP-1 site (TGAC/GTCA) which is normally bound by the proteins of the Jun/Fos family. The AP-1 consensus was enriched among Meis1-bound peaks in all cell lines but became the single enriched sequence in M and P cells (Figure 3A). OCTA was enriched among Meis1 peaks in WT, ev, and MP cells but the fraction of AP-1 containing peaks was generally higher; importantly, OCTA was not at all enriched in P or tumorigenic M cells (Figure 3A) in which, strikingly, only the AP-1 consensus was enriched. The OCTA consensus constituted 36% and 39% of the peaks in WT and ev cells and 21% in the MP cells (Table 1). OCTA sites were rarely present in the same peaks together with AP-1 (Figure 3A). The frequency of AP-1 consensus sites was very high among Meis1 peaks, 66% in ev, 43% in WT, 64% in P cells, 45% in MP and 56% in M cells (Table 1 and Figure 3A).

**Table 1:** Representation of AP-1 consensus sequences within Meis1 and Prep1 peaks

Protein	Cells	n*	Consensus sequence	n OCTA or DECA	n AP-1
Meis1	Ev	4,328	OCTA, AP-1	1,570 (36.3%)	2,854 (65.9%)
Meis1	WT	10,399	OCTA, AP-1	4,049 (39%)	4,474 (43%)
Meis1	P	3,473	AP-1	-	2,214 (64%)
Meis1	MP	12,008	OCTA, AP-1	2,498 (20.8%)	5,432 (45.2)
Meis1	M	27,284	AP-1	-	15,334 (56%)
**Prep1**	WT	2,949	DECA, AP-1	2,267 (77%)	466 (15.8%)
**Prep1**	MP	13,562	DECA, AP-1	9,882 (73%)	2,998 (22%)
**Prep1**	P	17,450	DECA, AP-1	12,528 (72%)	5,808 (33,3%)

* n: number of peaks containing either or both of the consensus sequences. Hence the total number of sequences may exceed 100%.

AP-1 sites were enriched much less among Prep1 peaks (Table 1), which featured DECA as the most enriched: 77% in WT, 72% in P and 73% in MP cells (Table 1). However, also the AP-1 consensus was enriched, 15.8% in WT, 22% in MP and 33% in P cells (Table 1 and Figure 3A). Overall, in MEFs Prep1 bound mostly DECA and, less, AP-1; however, Meis1 bound almost exclusively AP-1 in P and M cells or more AP-1 than OCTA in the other cells. Importantly, in MP cells the AP-1 sites decreased among Meis1 peaks with respect to M cells, indicating that Prep1 overexpression competes with Meis1 to prevent its binding to the AP-1 consensus.

An *a posteriori* search for OCTA, DECA and AP-1 motifs confirmed this result on all sequence sets using FIMO [14] with default parameters (Methods, Supplementary Figure S2).

The position of the OCTA and AP-1 consensus within Meis1 peaks was slightly different (Figure 3B). Among peaks containing a single enriched consensus, the OCTA site mostly coincided with the peak summit; AP-1, instead, was a little more spread at the two sites of the summit. However the M cells were a relevant exception since the search for a consensus sequence did not identify OCTA but an exclusive AP-1 site enrichment. The position of this sequence in this set of peaks was very close to or coincided with the peak summit much more often than in the other cells (Figure 3B).

**Figure 4:**
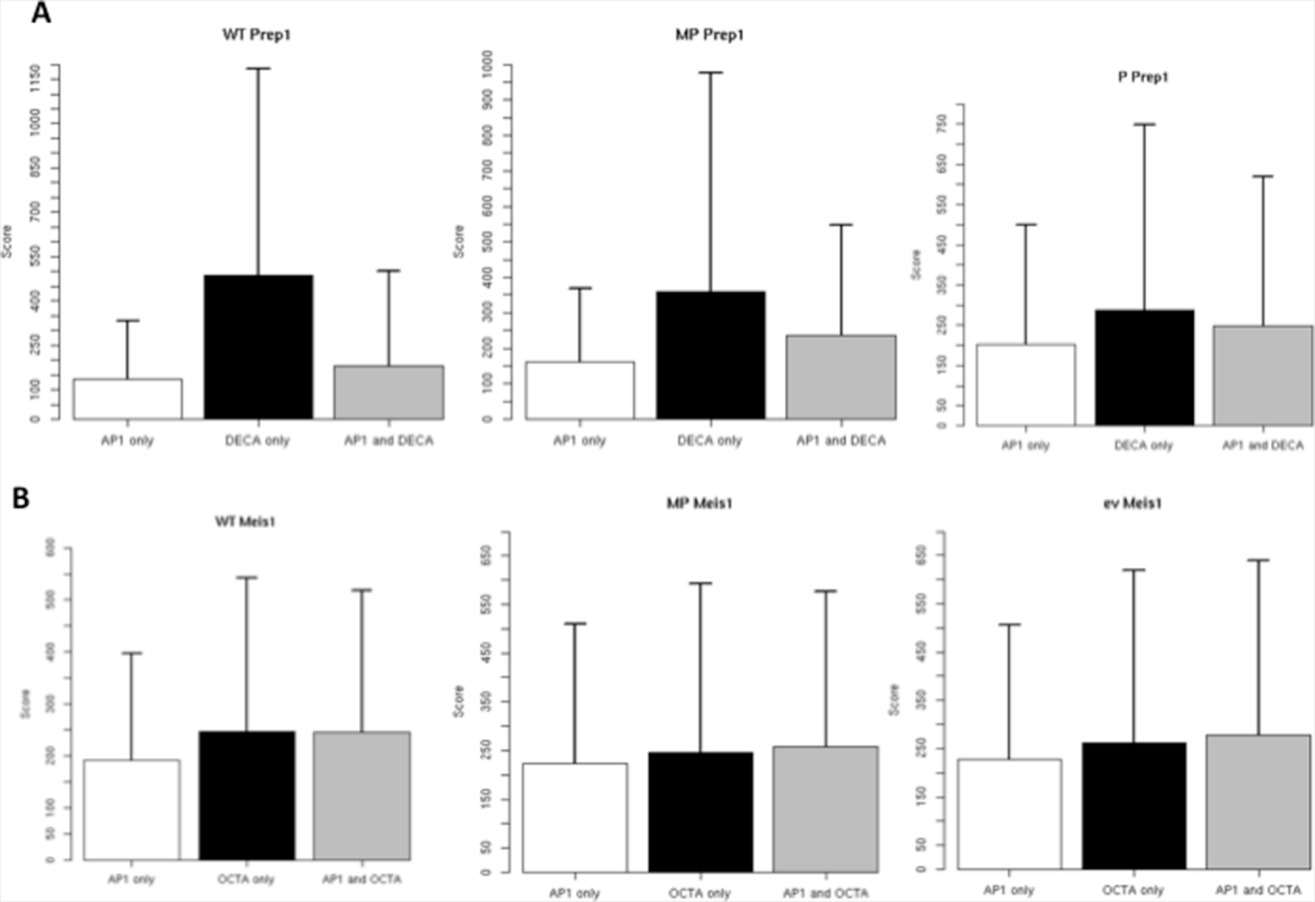
Differential score of peaks containing AP-1 and OCTA or DECA consensus in the different cell lines The score parameter derives directly from the difference between the number of ChIP-seq reads in the immuno-precipitated v. the input sample.

We next exploited the peak scores to compare the two consensus. A higher score does not directly indicate but must be related to higher affinity or binding to a higher fraction of the cells. In WT, P and MP cells, Prep1 peak score for DECA was on the average much higher than for AP-1 or DECA plus AP-1 consensus sites (Figure 4). No differences were instead observed for Meis1. The higher score of DECA for Prep1 may agree with Prep1 competing with Meis1 to the binding to AP-1 sites in MP cells.

### Meis1 and Prep1 indeed bind the AP-1 consensus *in vitro* and *in vivo*

**Figure 5:**
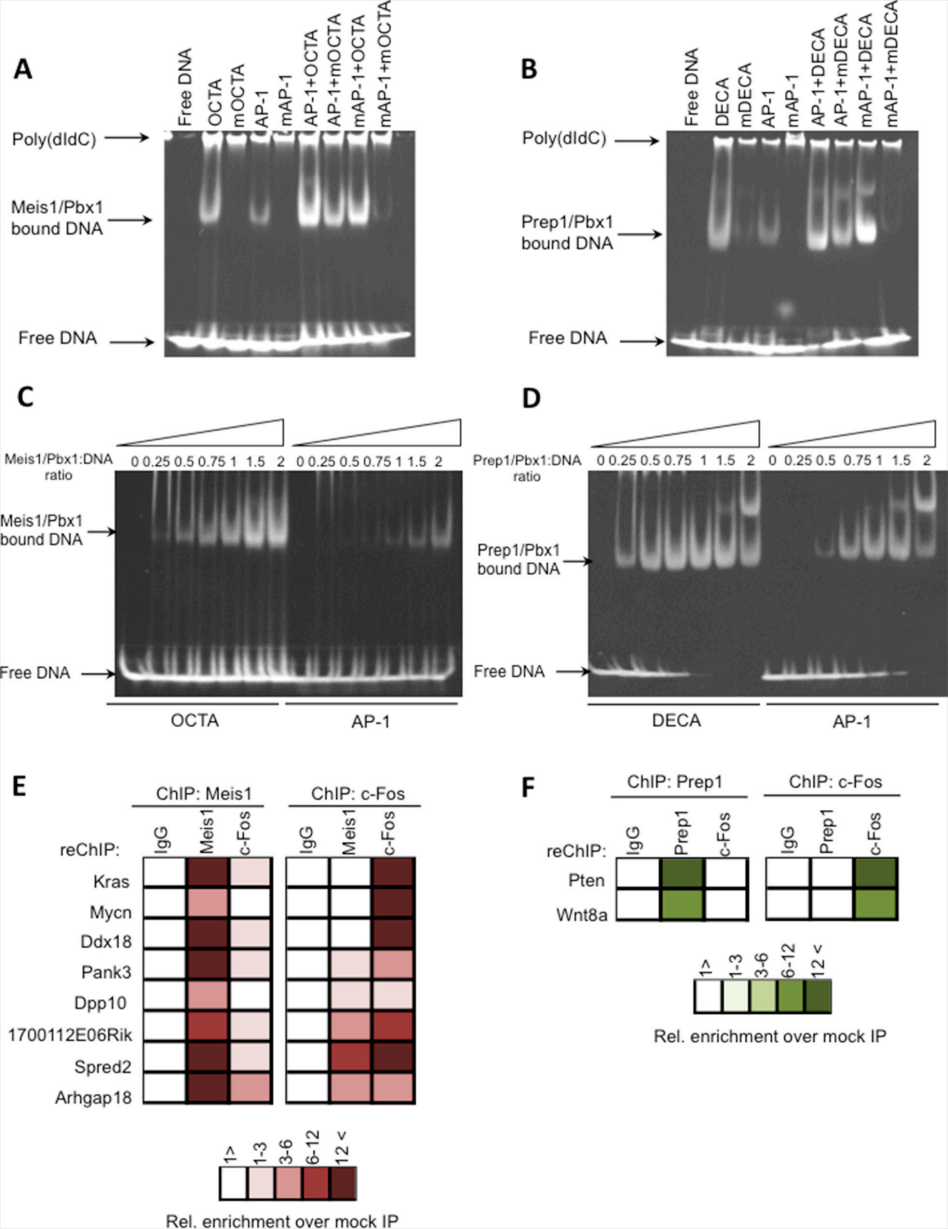
Meis1 and Prep1 bind specifically AP-1 sites EMSA analysis of DNA binding of recombinant Meis1-Pbx1 and Prep1-Pbx1 complexes. Recombinant proteins were incubated with the indicated oligonucleotides the sequence of which is shown in Supplementary Materials and Methods. The arrows to the left indicate the position of the various DNA-bound complexes as well as unbound DNA. **A.** EMSA analysis of DNA binding of recombinant Meis1-Pbx1 complexes to the AP-1 oligonucleotides indicated on the top. **B.** EMSA analysis of DNA binding of recombinant Prep1-Pbx1 complexes to the AP-1 oligonucleotides. **C.** Concentration dependent binding of the Meis1-Pbx1 dimer to the OCTA and AP-1 oligonucleotides indicated on the top. **D.** Concentration dependent binding of the Prep1-Pbx1 dimer to the DECA and AP-1 oligonucleotides. Slower-migrating complexes are probably due to the formation of recombinant protein oligomers. **E–F.** Eight genomic regions bound by Meis1 in M cells and two genomic regions bound by Prep1 in P cells were validated, using primers specific for the individual regions and listed in Supplementary Table S1. Q-PCR was used to determine the amount of precipitated DNA and the relative enrichment over the mock-precipitated input. (**E**) ChIP-reChIP analysis for Meis1-bound genomic regions in M cells. Chromatin was immunoprecipitated with either Meis1 or c-Fos antibodies and the immunoprecipitate was re-precipitated with Meis1, c-Fos or non-immune IgG, as color-coded. (**F**) ChIP-reChIP analysis for Prep1, c-Fos and IgG binding to the indicated genes in P cells. Chromatin was immunoprecipitated with Prep1 antibodies and the immunoprecipitated chromatin re-ChIPped with non-immune IgG, Prep1, c-Fos antibodies.

*In vitro* EMSA with a highly purified recombinant Meis1/Pbx1 heterodimer (see Methods) was used to show DNA binding to oligonucleotides containing OCTA, AP-1 or OCTA+AP-1 sites; likewise, the recombinant Prep1/Pbx1 heterodimer was tested with DECA, AP-1 or DECA+AP-1. The DNA sequences employed were chosen among the actual ChIP-seq peaks (see Methods in the Supplementary section), but a single nucleotide substitution abolished binding (Figure 5).

Meis1/Pbx1 complex specifically bound both OCTA and AP-1 sequences and Prep1/Pbx1 complex both DECA and AP-1 sequences (Figure 5A, 5B). They also specifically bound to combined OCTA-AP-1 or DECA-AP-1 oligonucleotides, respectively. This novel result supports the ChIP-seq indications. The functionality of the AP-1 sequence chosen was validated in EMSA with c-Fos/c-Jun complex (Supplementary Figure S3).

To test the relative affinity of Meis1/Pbx1 and Prep1/Pbx1 complexes for OCTA or DECA versus AP-1, we used EMSA at different protein:DNA ratios. Meis1/Pbx1 bound AP-1 at a higher protein:DNA ratio than OCTA (Figure 5C) whereas Prep1/Pbx1 bound AP-1 also at low protein:DNA ratios (Figure 5D).

Overall, these results suggest that Meis1 can indeed occupy AP-1 sites. Since in M cells AP-1 sites constitute the only enriched consensus, their binding by Meis1 must be connected to its oncogenic activity (Figure 3A). The higher affinity of Prep1/Pbx1 towards AP-1 is consistent with Prep1 inhibition of Meis1 tumorigenicity (MP cells): compared to M cells, Meis1 level was decreased 2.5-fold whereas Prep1 increased substantially (Figure 1B, 1C). This indicates that the competition of Meis1 and Prep1 for AP-1 binding sites may be involved in the tumor versus non-tumor decision.

Conventional ChIP validated the *in vivo* binding of Meis1 to the AP-1 site of eight Meis1 peaks harbouring only an AP-1 site and no OCTA. Meis1 was always immunoprecipitated from the tested genes in MEFs (Figure 5E), but re-chipping with c-Fos antibodies was mostly negative. Chipping with a c-Fos antibody was also positive but again re-chipping with Meis1 antibodies was negative (Figure 5E). Therefore Meis1 and c-Fos binding appeared to be mutually exclusive. The summit of the validated peaks and the primers used are shown in Supplementary Table S1.

Also *in vivo* binding of Prep1 and c-Fos to the AP-1 sites of *Pten* and *Wnt8a* (Figure 5E) was mutually exclusive.

### Specific target-gene sets are associated to Meis1 tumorigenesis or Prep1 tumor-suppression

Our five-cell lines system allows to identify the genes responsible for the competing activities of Meis1 and Prep1. Total RNA was extracted from each cell line and the experiment was carried out in duplicate for WT, P and MP cells; in triplicate for ev cells and in quadruplicate for M cells. The RNA was deep-sequenced and analyzed by RNA-seq (Supplementary Table S2). Sequencing generated 200–300 millions total raw reads per sample, of which about 25–50 millions were mapped and aligned, a fraction that is normally observed in RNA-seq experiments [15] The data from each experiment of the individual cell lines were combined and averaged out (available at GEO, accession number GSE58818). WT and ev cell lines were used as controls and the genes affected were subtracted from the subsequent analyses because independent of tumorigenesis (see below). Intersecting RNA-seq and ChIP-seq in tumorigenic M v. ev cells identified 1395 differentially expressed genes (*p* < 0.05%) of which 45% (*n* = 635/1395) were directly bound by Meis1 (Supplementary Figure S4A). Of the 635 Meis1 target genes, 81% (*n* = 515) were upregulated upon Meis1 overexpression, suggesting an activatory role for Meis1. Since the M cells are tumorigenic, these genes should include those directly involved in Meis1 tumorigenesis.

We also compared RNA-seq of MP and ev cells to identify the Meis1 gene targets in the presence of overexpressed Prep1. Of the 1216 differentially expressed genes in MP cells (Supplementary Figure S4B), 38% (448) were bound by Meis1. This represents a decreased percentage of Meis1 direct target genes, with respect to 45% of M cells (Supplementary Figure S4A). However, Meis1 still overall activated gene expression (Supplementary Figure S4B). Likewise, Prep1 also exerted an activatory role in MP cells, since 85% (381 genes) of the genes bound by Prep1 (488 genes) were upregulated (Supplementary Figure S4C).

**Figure 6:**
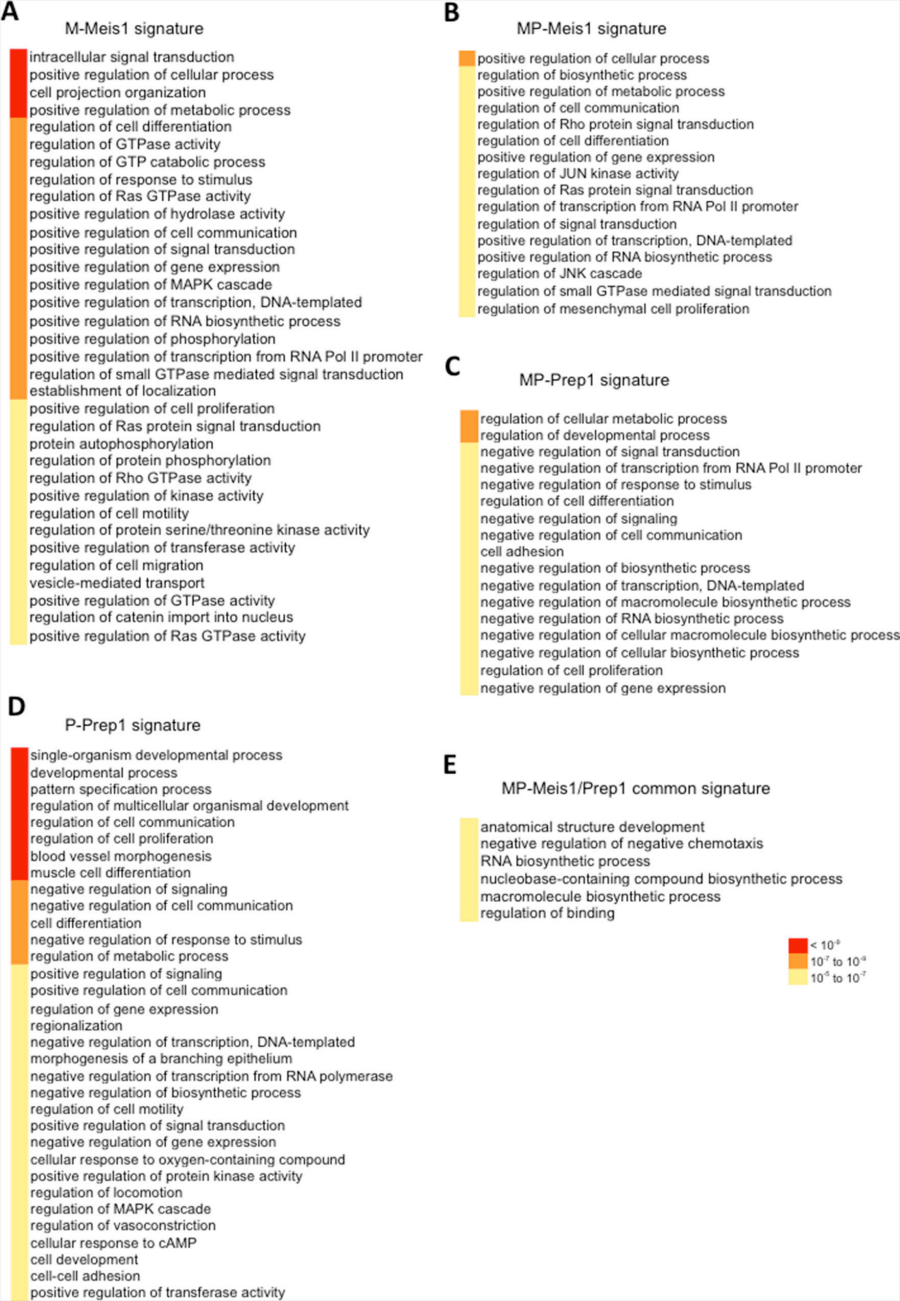
The Meis1 oncogenic and the Prep1 tumor-suppressive signatures Functional annotation of Meis1 and Prep1 cell-type specific signatures. Gene ontology terms (from GOrilla) enriched among genes bound and up-regulated by Meis1, Prep1 or both (common) in the indicated cell lines. The *p-value* ranges are color-coded.

We focused our attention on the upregulated gene sets. To identify a cancer-related Meis1 signature in M and MP cells, we subtracted from these sets the targets in common between M and WT (*n* = 111) (Supplementary Figure S4D) and between MP and WT (*n* = 116) (Supplementary Figure S4E). Likewise, we identified a tumor suppressive gene signature for Prep1 subtracting the targets in common between MP and WT and between P cells and WT (Supplementary Figure S4F and S4G). Moreover, we identified the targets up-regulated by both Prep1 and Meis1 in MP cells (Supplementary Figure 4H) and subtracted them from the Meis1 target set in M and from the Prep1 target set in P cells (Supplementary Figure 4I). This type of analysis resulted in the establishment of five gene signatures on which we performed Gene Ontology Analysis, which is reported in Figure 6. The list of genes in the various signatures is presented in Supplementary Table S3.

The analysis of Figure 6 shows in most signatures a statistically very significant enrichment of gene categories involved in signal transduction, transcription from PolII promoter, gene expression, cell proliferation, cell communication. Importantly, the comparison of the M-Meis1 (Figure 6A) and the MP-Prep1 (Figure 6C) signature evidentiated the same categories. However, the genes within these categories had opposite function in the two signatures: positive in Meis1 versus negative in Prep1.

A different result was observed comparing the M-Meis1 (Figure 6A) to the MP-Meis1 (Figure 6B) signature. In both cells the same categories were enriched, but their genes acted in the same direction, with a somewhat general decrease of the statistical significance in the MP-Meis1 cells. Prep1 signature in P (Figure 6D) versus MP (Figure 6C) cells, instead, showed both divergent as well convergent enriched categories. Finally, comparison of the M-Meis1 (Figure 6A) to the P-Prep1 signature (Figure 6D) again showed mostly divergent categories. Thus, interestingly, the enriched categories in The Meis1 and Prep1 signatures were largely shared, but the individuak genes acted in opposite directions in M v. MP cells.

In agreement with its oncogenic activity, the M-Meis1 signature (Figure 6A) was enriched for signaling pathways involved in cellular transformation such as Ras, Rho and MAPK pathways. Moreover, Meis1 positively regulated cellular proliferation and motility (Figure 6A). On the other hand, in less tumorigenic MP cells (Figure 6B), the statistical significance of the potential tumorigenic signaling pathways decreased. In these cells the Prep1 signature (Figure 6C) was specifically enriched in functions that negatively regulate cellular proliferation, RNA metabolic process, transcription and signal transduction. Thus Prep1 inhibition of Meis1 combines a dampening of Meis1 activity to the activation of pathways opposing Meis1 functions in M cells. Moreover, the Prep1 signature shifted from developmental processes in P cells (Figure 6D) to suppression of signal transduction and transcription in MP cells (Figure 6C). Finally, the Meis1/Prep1 common signature in MP cells showed enrichment in the developmental and biosynthetic processes, but at low statistical significance (Figure 6E).

The above results are significant as underlined by Gene Set Enrichment Analysis. This identifies with high significance 51 genes of the M-Meis1 signature whose expression is down-regulated upon inhibition of the EGF-R (*p* = 2.13e^−24^) in differentiating normal cells, and 7 genes (*p* = 9.3^e-6^) upregulated in human lung cancer cells that overexpress a mutated form of Kras (Supplementary Table S4).

In the Meis-1 ChIP-seq peaks of most cells the position of the AP-1 consensus sequence only partly coincided with the summit of the peak, the only exception being the tumorigenic M cells (Figure 3B). Therefore, to better substantiate that Meis-1 tumorigenic activity was connected to the binding to an AP-1 site, we performed Gene Ontology analysis on the subset of 121 genes in which the position of the AP-1 consensus coincided with the peak mid-point (+/− 30bp). We only chose peaks that did not contain any OCTA consensus sequence. Supplementary Table S5 shows that among the Gene Ontology categories enriched in this analysis, many of the same observed in the M-cells Meis1 signature of Figure 6 appeared. In this subset, their degree of enrichment became even more significant. Therefore, the Gene Ontology results obtained by either selecting Meis1 peaks containing only AP1 sites coinciding with the peak summit (Supplementary Table S5) or Meis1 peaks having tumorigenic relevance (Figure 5), yielded the same result. This data reinforces the idea that the tumorigenic activity of overexpressed Meis1 depends on, or importantly involves, its binding to AP-1 sites and hence that Meis1 selects certain genes normally controlled by the Jun/Fos protein family.

## DISCUSSION

We have analyzed the importance of dysregulation of oncogenes and tumor suppressors expression. Prep1 and Meis1 have opposing functions in tumorigenesis, respectively as tumor suppressor [6, 8, 10, 16] and oncogene [5, 10, 17]. In the MEF system neoplastic transformation depends on the absolute level of Meis1 and by the absence of Prep1 [10]. This translates into the activation of a different gene expression program.

The present genome-wide analysis provides a series of important unexpected information. Since the expression of *Prep1* and *Meis1* is heavily affected in a very large percent of human cancers [6, 18, 19], the present data are of general pertinence and interest.

The number of genes bound by Meis1 and Prep1 in the five cell types correlates with their level of expression (Figure 1D), leading to occupancy of new target genes, beyond a core set bound in the WT and ev cells. In this feature, Meis1 and Prep1 are similar to MyoD, Oct4 and Sox2 transcription factors that when overexpressed bind new target genes [20]. However, Meis1 and Prep1 differ from c-Myc which at high levels of expression in tumor cells does not enlarge its set of target genes, but rather occupies more enhancers activating their transcription [1].

In the E11.5 mouse embryo or in ES cells, Prep1 and Meis1 occupy mostly TSS-associated and TSS-remote regions, respectively [11]. Instead, in WT MEFs Prep1 is 10-fold less abundant at TSS regions than in the embryo, although still 4-fold more than Meis1. However, Prep1 level is elevated (P and MP cells) it tends to increase its binding (7-fold) to enhancers, whereas Meis1 maintains the same distribution (compare WT and M cells) among genomic regions as in the embryo trunk. The increased binding of Prep1 to enhancers represents a novel Prep1 function, since while promoters determine gene expression, enhancers are involved in their regulation.

### Prep1 and Meis1 bind AP-1 sites in MEFs

Unexpectedly, the DNA consensus sequence also bound by Prep1, but mainly Meis1, is the AP-1 consensus sequence (TGATCAG) which, like the OCTA consensus, is often very close to the peaks summit (Figure 3B). Binding to the AP-1 site was specific, but the Prep1-Pbx1 complex had a higher affinity than Meis1-Pbx1. Meis1 and c-Fos bound 8/8 and Prep1 and c-Fos 2/2 of the tested endogenous gene sequences within the identified peaks (Figure 5), but they were never together on the same gene. Therefore it appears that, at least for the analyzed genes, the binding of Meis1 or Prep1 to the AP-1 sites is mutually exclusive.

Binding to AP-1 may have an important function in Meis1 oncogenic activity (M cells), because AP-1 becomes the main if not the only consensus sequence for Meis1 binding. Moreover, the inhibition of Meis1 tumorigenicity by co-overexpression of Prep1 (MP cells) correlates with the decrease of the number of the “extra” Meis1 targets, not the core genes, hence of those containing the AP-1 binding sites. The presence of AP-1 sites also among the Prep1-bound sequences is in line with the transcriptional competition between the two transcription factors [10]. Indeed, 40.8% of the Meis1 extra peaks lost in the MP cells are in fact bound by Prep1. Prep1 competition may rely on the higher affinity for the AP-1 DNA (Figure 4). Indeed, Prep1-Pbx1 *in vitro* binding affinity to AP-1 is higher than Meis1-Pbx1 (Figure 5D).

### Meis1 oncogenic and Prep1 tumor-suppressor signature

The overlap of ChIP-seq and RNA-seq data has allowed to extract from the totality of their target genes a cancer-specific Meis1 and Prep1 signature (Figure 6). In this analysis we consider only those genes not only bound by Prep1 or Meis1 but also affected by their overexpression in M and MP cells. Strikingly, in both signatures the same gene categories are enriched, however the genes included in these categories regulate the pathways positively in the case of Meis1 and negatively in the case of Prep1.

Overexpression of Meis1 and to a lesser extent of Prep1 preferentially leads to up-regulation of their target genes. We have therefore considered only those genes whose expression is enhanced by either Meis1 or Prep1. With these caveats, we consider the genes bound and overexpressed by Meis1 in M cells as potentially tumorigenic and those by Prep1 in MP cells as potentially tumor suppressive. Thus, Meis1 oncogenic signature, M-Meis1 signature (Figure 6A), includes genes with functions relevant to cellular transformation. On the other hand, the Prep1 signature in the less tumorigenic MP cells, MP-Meis1 signature, includes genes that down-regulate or inhibit the same functions (Figure 6B). Thus the decrease of Meis1 level induced by Prep1 (see Figure 1) is coupled to the inhibition of the oncogenic mode of the cell metabolism.

The importance of the AP-1 sites in Meis1 tumorigenicity was reinforced by the Gene Ontology analysis performed on the genes containing uniquely an AP-1 site in the peak sequence, and in which the AP-1 site coincided with the peak summit.

## MATERIALS AND METHODS

### ChIP-seq, ChIP re-ChIP

ChIPs and ChIP re-ChIPs were carried out on various cell-types using standard methods [11]. The details are provided in Supplementary Materials and Methods.

### Data analysis

All ChIPseq data set were mapped to the mouse (mm9) genome using Bowtie 1.0 software [21]. The alignments were used for peak calling using MACS (1.4) algorithm [22]. Peaks with a *P*-value cutoff of 10^−6^ and a false discovery rate of < 5% were selected for subsequent analysis. The sets of peak coordinates for Meis1 or Prep1 in one cell-type were intersected with the sets for Meis1 or Prep1 in the other cell-types. Two peaks were considered overlapped if they showed 50% or more overlapping sequences. Venn diagrams were generated using BioVenn software [23].

RNAseq data sets were aligned to mouse (mm9) genome using default parameters of TopHat [24]. Cufflinks was used to assemble mapped reads into iso-forms. Biological replicates were merged using Cuffmerge. Differential expression analysis was performed using Cuffdiff, and differentially expressed genes were visualized using CummeRbund [24]. *P*-value cutoff of 0.05 was used. Intersects between ChIPseq and RNAseq data were performed on Galaxy platform.

### Gene ontology analysis

Gorilla was used to assess gene ontology terms comparing the lists of genes bound and regulated by Meis1 or Prep1 to the list of genes in Ensembl v63 with a *P*-value cutoff of 10^−4^.

### Recombinant Meis1-Pbx1 and Prep1-Pbx1 preparations

Details of cloning, expression and purification of recombinant proteins used in EMSAs are described in Supplementary Materials and Methods.

### EMSA

EMSA reactions were performed following the standard protocols. Details of the reactions are provided in Supplementary Materials and Methods.

## SUPPLEMENTARY MATERIALS AND METHODS: FIGURES AND TABLES




