# Supplementary material for: Tumorigenesis by Meis1 overexpression is accompanied by a change of DNA target-sequence specificity which allows binding to the AP-1 element
Source: Oncotarget. 2015 Aug 3;6(28):25175–87. doi: 10.18632/oncotarget.4488 (PMC4694823; doi:10.18632/oncotarget.4488)
Supplement: Supplementary file 1 [file oncotarget-06-25175-s001.pdf]

SUPPLEMENTARY MATERIALS AND METHODS

Cell culture and viruses

Passage-35 *Prep1*$^{i/i}$ and WT MEFs [1] were cultured in DMEM supplemented with 10% heat-inactivated fetal bovine serum, 100 U/mL penicillin, 100 μg/mL streptomycin and 2 mM L-glutamine at 37°C in 5% CO_2. pMSCVpuro-*Meis1a* and pMSCVhygro-Prep1 retroviral expressing vectors have been described [1]. Meis1a and Prep1 carrying retroviruses were produced in phoenix-eco cell line and used for infection of *Prep1*$^{i/i}$ and WT MEFs. Infected cells were selected with 2 μg/mL of puromycin and/or 100 μg/mL of hygromycin.

Western blotting

Western blot analysis was performed following standard procedures. 50 μg of the total cell extracts from indicated cell-types were separated in a 10% SDS-polyacrylamide gels using electrophoresis. The following antibodies were used to detect the proteins of interest: anti-Meis1 (kindly provided by M. Torres, CNIC, Madrid, Spain), anti-Prep1 (sc-6245x, Santa Cruz) and anti-vinculin (V9B1, Sigma-Aldrich) antibodies. The ImageJ64 software was used to normalize the results.

Cloning, expression and purification of recombinant proteins

DNA sequences encoding Prep1, Pbx1 and Meis1 were amplified by PCR with the following primers containing BamHI and XhoI restriction sites:

Prep1 forward 5′- cgcggatccatgatggctacacagacatta agtatag-3′

Prep1 reverse 5′-ccgctcgagttacctctgaactggccggttc-3′

Pbx1 forward 5′-cgcggatccatggacgagcagcccagg-3′

Pbx1 reverse 5′- ccgctcgagttatttggcagcataaatattgg cttc-3′

Meis1 forward 5′-cgcggatccatggcgcaaacgtacga-3′

Meis1 reverse 5′-ccgctcgagttacatgtagtgccactgcc-3′.

The amplified PCR products were inserted in a modified pGEX6p vector (pGEX6p-2rbs) via BamHI and SalI restriction sites. The mutated DNA was transformed into competent TOP10 *E. coli* cells (Invitrogen). The protein inserts were validated by DNA sequences.

Protein expression in the *E. coli* strain BL21(DE3) pLysS (Promega) was induced with 0.1 mM isopropyl-ß-D-thiogalactopyranoside (IPTG). Expression was continued for 16 h at 16°C. Cells were harvested by centrifugation at 4,000 rpm for 15 minutes in a Beckman JLA rotor and resuspended in 20 mL lysis buffer (50 mM Tris pH 7.4, 1 M NaCl, 10% glycerol, 5 mM DTT, supplemented with Protease Inhibitor Cocktail Set from Calbiochem). Sonication was done with a Bandelin Sonopuls sonicator for 3 × 45 seconds with 5 pulses at 30–40% of max power. After sonication, bacterial lysates were cleared by centrifugation at 40,000 × g for 2 hours using a Beckman JA-20 rotor. The GST-fused proteins were purified using glutathione-agarose beads (GE Healthcare). After 2 hours of agitation at 4°C, beads were first washed with 10 volumes of 50 mM Tris pH 7.4, 0.5 M NaCl, 10% glycerol, 5 mM DTT followed by 20 volumes of 50 mM Tris pH 7.4, 0.3 M NaCl, 10% glycerol, 5 mM DTT. The protein complex was cleaved from GST with 10 μg/mL of Protease 3C for 16 hours at 4°C.

EMSA

Purified recombinant Meis1/Pbx1 and Prep1/Pbx1 heterodimers were prepared as mentioned above. The ability of these complexes to bind to the identified consensus motifs *in vitro* was tested by EMSA. Assays were performed in 15 μL reaction mix containing 10 mM Tris-HCl (pH 7.4), 100 mM NaCl, 1 mM DTT, 5% glycerol, 1 μg poly-dIdC, 1.3 μM double-stranded oligonucleotide and 12 μg of Meis1/Pbx1 or 7 μg of Prep1/Pbx1 complexes. For the EMSA using c-Fos/c-Jun heterodimers, 1.5 μg of each protein was mixed together and incubated on ice for one hour before adding the oligonucleotides. Reactions were carried out for 30 min on ice, and the complexes resolved by 5–15% non-denaturing PAGE. The gels were stained with GelRed (Biotium) to visualize oligonucleotides.

The following double-stranded oligonucleotides along with their mutated versions were used. "m" in front of motif name stands for mutant. The corresponding sequence for each motif within the oligonucleotide sequences is underlined. These sequences were taken either from Meis1 or Prep1 target peaks.

5′- GAAAGTGATAAATAATACAAGGAAAGC -3′ (Meis1 peak)-OCTA

5′- GAAAGTACCAAATAATACAAGGAAAGC -3′ (Meis1 peak)-mOCTA

5′-CATTACCATGAGTCATTGGGCTTGCTC-3′ (Meis1 peak)-AP1

5′-CATTACCATGCTCCATTGGGCTTGCTC-3′ (Meis1 peak)-mAP1

5-ATTATAGTGATGAATCATCATGACTCATGG TCCAG-3′ (Meis1 peak)-OCTA+AP1

5-ATTATAGTACCGAATCATCATGACTCATGG TCCAG-3′ (Meis1 peak)-mOCTA+AP1

5-ATTATAGTGATGAATCATCATGCTCCATGG TCCAG-3′ (Meis1 peak)-OCTA+mAP1

5-ATTATAGTACCGAATCATCATGCTCCATGG TCCAG-3′ (Meis1 peak)-mOCTA+mAP1

5′-AGCTGACCTCTGAGTGACAGGGTGGAAT GTGA-3 (Prep1 peak)-DECA

5′-AGCTGACCTCTGCGTGCAAGGGTGGAAT GTGA-3 (Prep1 Peak)-mDECA

5′-TAAAATGCTGAGTCACTGACTGACAGCTT TAGGCT-3′ (Prep1 peak)-AP1+DECA

5′-TAAAATGCTGGTTCACTGACTGACAGCTT TAGGCT-3′ (Prep1 peak)-mAP1+DECA

5′-TAAAATGCTGAGTCACTGCCTGCAAGCTT TAGGCT-3′ (Prep1 peak)-AP1+mDECA

5′-TAAAATGCTGGTTCCCTGCCTGCAAGCTT TAGGCT-3′ (Prep1 peak)-mAP1+mDECA.

Recombinant c-Fos and c-Jun proteins were from Active Motif (Carlsbad, CA) Catalog No: 31115 and 31116, respectively.

Chromatin immunoprecipitation (ChIP) and Illumina sequencing

ChIPs were carried out on various cell-types using standard methods [2]. The details are provided in supplementary materials and methods. Briefly, 1% formaldehyde cross-linked chromatin was sonicated to generate 200–350 bp chromatin fragments and immunoprecipitated overnight at 4°C with 10 µg of anti-Meis1/2 (sc-10599x) and anti-Prep1 (sc-6245x) antibodies. 10 ng of purified dsDNA from both input and IP was used for library preparation following the Illumina TruSeq DNA Sample Preparation v2 kit protocol. Libraries were sequenced on Illumina HIseq-2000G to obtain 50 bp single-end reads.

For individual sites ChIP-reChIP, the chosen sites and the primers are described in Figure 5 and in Supplementary Table S2.

RNA isolation and mRNA-seq library generation

Total RNA was extracted from various cell-types using the Qiagen easy RNA kit. Illumina multiplexed sequencing libraries were prepared according to the manufacturer’s instructions. Libraries were sequenced using the paired-end 50 bp protocol.

Establishment of Meis1 and Prep1 gene signatures

Since in the present system Meis1 and Prep1 have mainly an activatory role, we focused our attention on the upregulated gene sets. To identify a cancer-related Meis1 signature in M and MP cells, we needed to subtract from the upregulated gene sets those Meis1 targets which were in common between M and WT (Supplementary Figure S5D) and between MP and WT cells (Supplementary Figure S5E). Subtraction of the common genes between M and WT (n = 111) and between MP and WT (n = 116) target sets resulted in 404 and 241 genes in M and MP, respectively (Supplementary Figure S5D and S5E). Likewise, we subtracted from the Prep1 target set the genes upregulated with respect to ev and in common between WT and MP (n = 67, Supplementary Figure S5F) or between WT and P (n = 89, Supplementary Figure S5G).

Since MP cells co-express Meis1 and Prep1, we defined three different target set categories: 1- genes which were bound and regulated by either Meis1 or Prep1; 2- genes which were co-bound by Meis1 and Prep1 but regulated only by one of them; 3- genes which were co-bound and co-regulated by both Meis1 and Prep1. The first and second category built up a Meis1- or Prep1-specific target set in MP cells. The last category was considered a common Meis1/Prep1 signature in MP cells. To identify the genes to be included in these categories, we overlapped Meis1 and Prep1 target sets (obtained from Supplementary Figure S5E and Supplementary Figure S5F) in MP cells (Supplementary Figure S5H). The genes which were only bound by Meis1 (n = 61) or Prep1 (n = 134) were included in a Meis1-specific or Prep1-specific first category. Those that were bound by both Meis1 and Prep1 (n = 180) were further overlapped with the Meis1 and Prep1 target sets in M and P cells, respectively, to distinguish between genes of the second and third categories. The MP cells genes bound by both Meis1 and Prep1 and that overlapped with those in either M (n = 36 plus 17) or P (n = 13 plus 17) cells were included in the Meis1-specific and Prep1-specific second category (Supplementary Figure S5I). Indeed they were regulated by either Meis1 in the absence of Prep1 (M) or by Prep1 in Meis1 low-expressing (P) cells. Thus in MP cells, although these genes are bound by both Meis1 and Prep1, they are most probably regulated by one of them. Therefore, we identified in these cells 114 Meis1-specific (n = 61 + 36 + 17) and 164 Prep1-specific genes (n = 134 + 13 + 17). The remaining 114 genes, which did not overlap with M or P signatures, were called Meis1/Prep1 common signature of MP cells.

REFERENCES

1. Dardaei L, Longobardi E, Blasi F. Prep1 and Meis1 competition for Pbx1 binding regulates protein stability and tumorigenesis. Proc Natl Acad Sci U S A. 2014; 111:E896–905.

2. Penkov D, San Martin DM, Fernandez-Diaz LC, Rossello CA, Torroja C, Sanchez-Cabo F, Warnatz HJ, Sultan M, Yaspo ML, Gabrieli A, Tkachuk V, Brendolan A, Blasi F, Torres M. Analysis of the DNA-Binding Profile and Function of TALE Homeoproteins Reveals Their Specialization and Specific Interactions with Hox Genes/Proteins. Cell Rep. 2013; 3:1321–1333.

SUPPLEMENTARY FIGURES AND TABLES

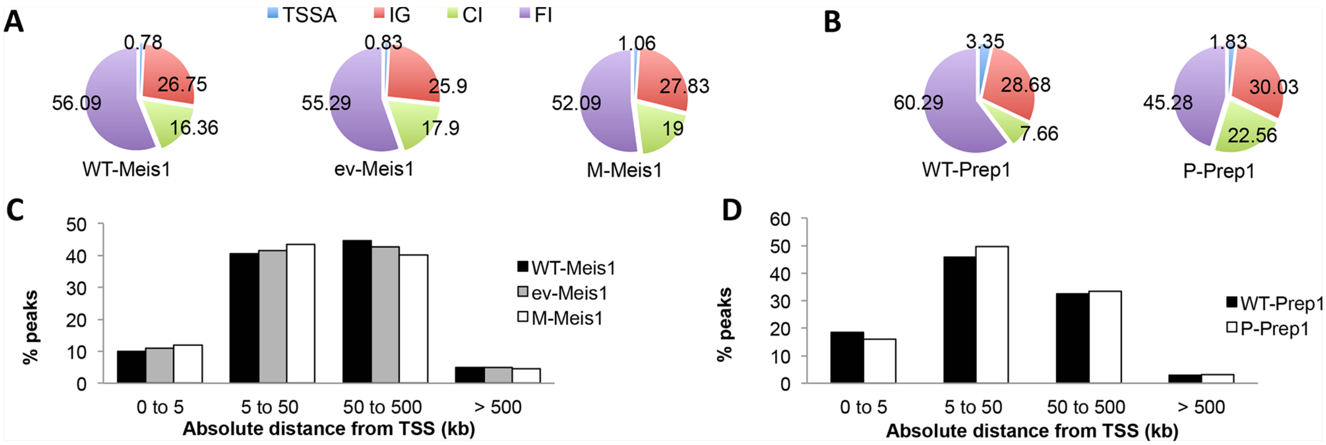

Supplementary Figure S1: Genomic distribution of Meis1 and Prep1 peaks in different cell lines. **A.** and **B.** Pie charts show overview of genomic distribution of Meis1 and Prep1 peaks in transcription-start-site-associated (TSSA), intragenic (IG), close intergenic (CI) and far intergenic (FI) in different cell types. **C.** Distribution pattern of Meis1 peaks with respect to TSS in WT, ev and M cells. **D.** Distribution pattern of Prep1 peaks with respect to TSS in WT and P cells.

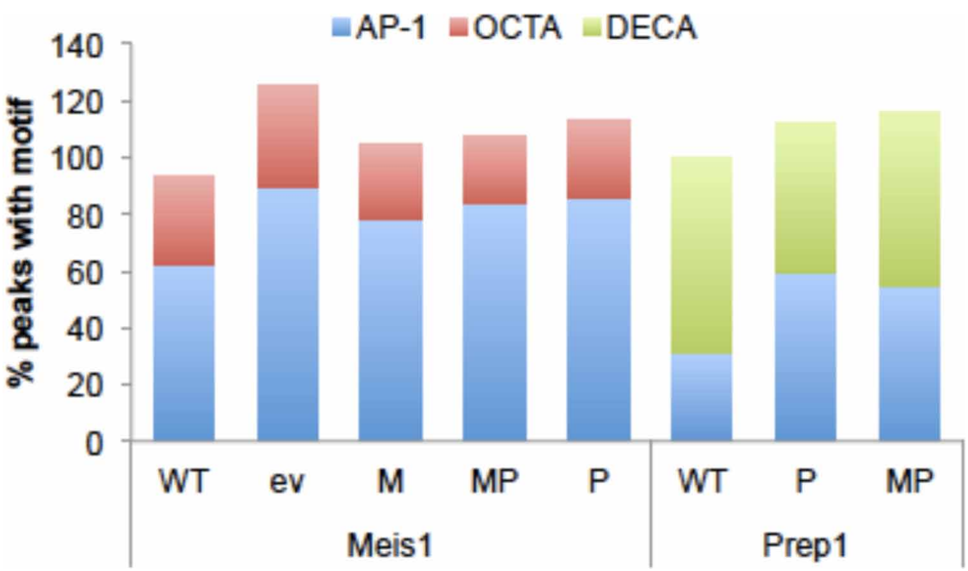

Supplementary Figure S2: Abundance of OCTA, DECA and AP-1 motifs identified by FIMO in different cell-types. *A posteriori* search for the OCTA, DECA and AP-1 motifs identified by rGADEM on all the sequence sets using FIMO with default parameters (see Supplementary Materials and Methods).

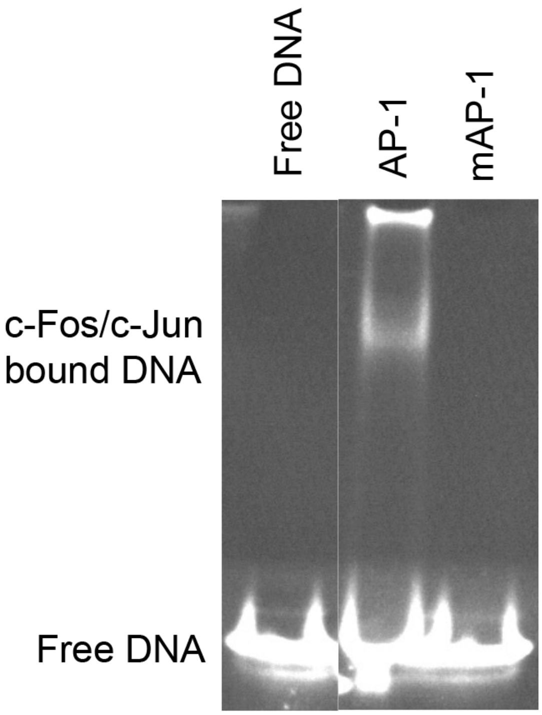

Supplementary Figure S3: Control EMSA analysis of recombinant c-Fos/c-Jun complex binding to the AP-1 and AP-1-mutated (mAP-1) oligonucleotide. The position of the bound complex and of unbound DNA are indicated.

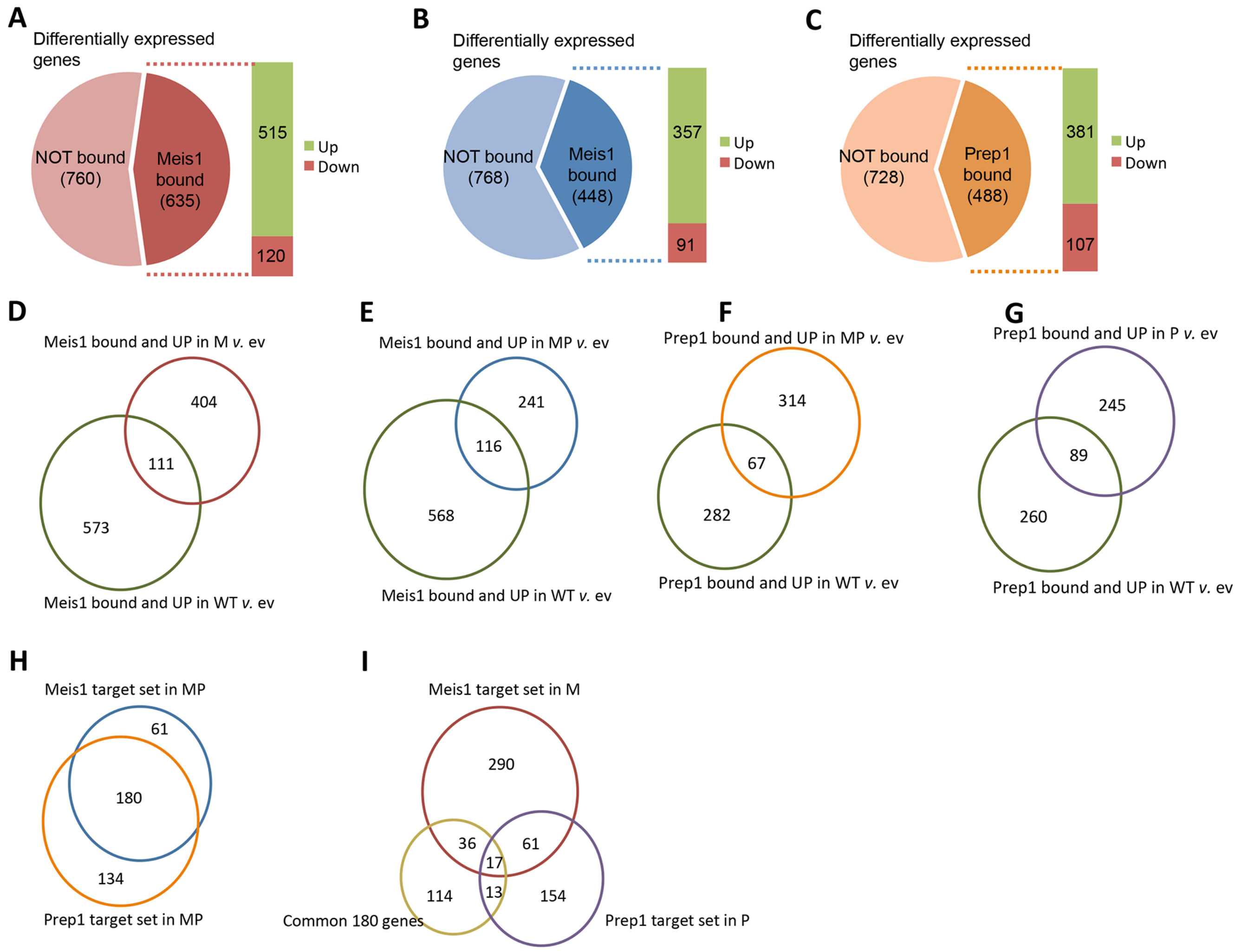

Supplementary Figure S4: Identification of Meis1 and Prep1 core and cell-type specific signatures. The differentially expressed genes that were also bound by Meis1 or Prep1 were obtained by intersecting ChIP-seq and RNA-seq results. Differential expression is related to the levels in ev cells. **A.** and **B.** Meis1-bound and not bound differentially expressed genes in M (A) and MP (B) cells. Stacked columns show the relative frequency of Meis1-bound target genes up-regulated (Up) and down-regulated (Down). **C.** Prep1-bound and not bound differentially expressed genes in MP cells. Stacked column shows the relative frequency of up-regulated (Up) and down-regulated (Down) Prep1-bound target genes. **D.** and **E.** Venn diagrams of upregulated Meis1 target genes in M versus WT (D) or MP versus WT (E) cells. **F–G.** Venn diagrams of upregulated Prep1 in MP versus WT (F) and P versus WT (G) cells. **H.** Venn diagrams showing the Meis1 and Prep1 target genes in MP. **I.** Venn diagrams showing the Meis1 target genes in M, Prep1 target genes in P cells and the common genes (the last taken from panel H).

Supplementary Table S1: List of the peaks, associated genes and primers used for ChIP-Re-ChIP*

Gene	Peak summit	Distance to TSS	Peak number	Primer sequence
Kras	Chr6 145322286	–72231	M25434	FWD: TGTATTGCTGCTCCAACAGG<br>REV: CACCATGGTCTTCTTCCCAT
Mycn	Chr12 12990120	–47977	M5558	FWD: TGAGTCACTAAAGCCAGTCCAC<br>REV: ATGACTTGAGCGCAGGAGTT
Ddx18	Chr1 121951804	–384089	M913	FWD: GCAGTTGCGTAGATCCATGA<br>REV: CACCACAGCTAGAATGGCAA
Pank3	Chr11 35652630	–117433	M3928	FWD:CGGGGACTTGTGACTCAGTA<br>REV:AACATCCCAGCTCTCTCTGC
Dpp10	Chr1 123294937	750119	M938	FWD: GTCAGTGATGAATGGCCAGA<br>REV: AATCATCACACTGGGGCTTC
1700112E06Rik	Chr14 22686169	666231	M8477	FWD: AATGGCTTGGTGAGTTTTGC<br>REV: ACCACTGGCATGGAACTCTC
Spred2	Chr11 19743345	–181041	M3747	FWD: GAGCTTCAAAAGATGCCACC<br>REV: CTGGCTGTGATTACCCACCT
Arhgap18/Lama2	Chr10 26969523	147027	M2200	FWD: TCAAAGACGTCAAGTGCCTG<br>REV: GGTTCAACTTGTTTTGGTGGTT
Pten	Chr19 32750465	19397	P8585	FWD: TGAGCCTGGCTTTCTCTAGG<br>REV: CCTGTCTGGCAGTCTGTGAC
Wnt8a	Chr18 34531645	–10749	P7833	FWD: GTTGTCAGACTTGGGGCAAT<br>REV: GGCTGGGAACTCGATTTGTA

*The results of the validation are shown in Figure 5E, 5F.

Supplementary Table S2: Number of mapped reads before and after removal of PCR duplicates

Sample Name	N. of raw reads	N. of reads mapped after removal of PCR duplicates
EV1	226,574,446	39,922,569
EV2	201,397,862	42,291,646
EV3	238,700,634	45,260,747
EV4	246,231,804	52,618,413
M1	221,265,434	39,665,658
M2	272,978,728	40,766,523
M3	328,266,792	51,616,454
M4	260,862,198	45,656,172
WT1	256,848,744	45,056,277
WT2	312,164,576	56,952,252
MP1	199,889,646	24,292,073
MP2	195,138,218	26,003,133
P1	270,661,620	49,193,717
P2	260,482,156	49,955,740

Supplementary Table S3: List of genes in the various signatures reported in figure 4

Supplementary Table S4: Gene set expression analysis of the Meis1 M-signature*

Gene set & Name	Description	*N* Genes in overlap	*p*-value	FDR *q*-VALUE
GOBERT_OLIGODENDROCYTE_DIFFERENTIATION_DN[1080]	Genes down regulated during oli-Neu differentiationin response to EGF-R inhibitorPD174265	51	2.13^{e-24}	7.25^{e-21}
GOZGIT_ESR1_TARGETS_DN[781*]	Genes down-regulated in ESR1-negative TMX2-28 breast cancer cell comparedto ESR1-positive MCF7	36	3.98^{e-17}	2.16^{e-14}
TGFB_UP.V1_UP [192]	TGB1-upregulated genes in20 epithelial cell lines.	20	2.4^{e-20}	4.63^{e-18}
P53_DN.V1_UP [194]	Genes up-regulated in NIH-60 Panel of cell lines with mutated TP53.	12	4.9^{e-10}	4.6^{e-8}
KRAS.LUNG_UP.V1_UP [141]	Genes upregulated in epithelial lung cancer cell linesOverexpressing an oncogenicForm of Kras.	7	9.3^{e-6}	1.96^{e-4}

The Meis1 M- and the Prep1 MP- signature gene sets have been overlapped with the Broad Institute GSEA database (http://www.broadinstitute.org/gsea/msigdb/index.jsp) comparing it with the Chemical and Genetic Perturbations (CGP) as well as the oncogenic signature set.

Supplementary Table S5: Gene ontology analysis of the M-cells peaks containing an AP-1 consensus at the summit

GO term	Description	*P*-value	FDR *q*-value
GO:0009893	positive regulation of metabolic process	2.3E-10	1.53E-6
GO:0010628	positive regulation of gene expression	4.58E-9	7.63E-6
GO:0048522	positive regulation of cellular process	3.37E-8	3.2E-5
GO:0045944	positive regulation of transcription from RNA polymerase II promoter	3.92E-8	3.48E-5
GO:0045893	positive regulation of transcription, DNA-templated	1.48E-7	7.59E-5
GO:1902531	regulation of intracellular signal transduction	1.52E-7	7.23E-5
GO:1902680	positive regulation of RNA biosynthetic process	1.55E-7	7.09E-5
GO:0048583	regulation of response to stimulus	2.32E-7	9.38E-5
GO:1902533	positive regulation of intracellular signal transduction	4.9E-6	9.45E-4

"*P*-value" is the enrichment *p*-value computed according to the mHG (minimum HyperGeometric) model, implemented in GOrilla.
"FDR q-value" is the correction of the *p*-value for multiple testing using the Benjamini-Hochberg method.
