# Supplementary material for: Tumorigenesis by Meis1 overexpression is accompanied by a change of DNA target-sequence specificity which allows binding to the AP-1 element
Source: Oncotarget. 2015 Aug 3;6(28):25175–87. doi: 10.18632/oncotarget.4488 (PMC4694823; doi:10.18632/oncotarget.4488)
Supplement: Supplementary file 2 [file oncotarget-06-25175-s002.pdf]

Supplementary Table S3: LIST OF GENES IN THE VARIOUS SIGNATURES REPORTED IN FIGURE 4.

| M-Meis1 (404 genes) | MP-Meis1 ( 114 genes (61+36+17) | MP-Prep1 ( 164 genes)) (134+13+17) | MP-Meis1/Prep1 common (114 genes) | P-Prep1 (245 genes) |
|---|---|---|---|---|
| Med15 | Pard3b | Eya1 | Hs6st1 | Ralgps2 |
| Hdac4 | Kif14 | Igfbp5 | Xpr1 | Igfbp5 |
| Slc6a17 | Pou2f1 | Pax3 | Ccdc93 | Pax3 |
| Tmem144 | Rab23 | Fzd5 | Mreg | Per2 |
| Nod1 | Gpr161 | Per2 | Lpgat1 | Epha4 |
| Ntrk3 | Mpzl1 | Fam126b | Zfp281 | Wnt10a |
| Zbtb40 | Cry1 | Wnt10a | Pbx1 | Ankrd44 |
| Daam1 | Pde7b | Ercc5 | Nrbf2 | Lss |
| Pard3b | Pcnt | Eif5b | 2610008E11Rik | Perp |
| Ptk2b | Hlf | Syt1 | Cdk19 | Map3k5 |
| Cyp26b1 | Rel | Helb | Nuak1 | Gria1 |
| Slco3a1 | Abca5 | Aim1 | Slc35d3 | Abr |
| Pld1 | Myo18a | Tbc1d15 | Chst11 | Mpp2 |
| Rorb | Plekhm1 | Shc2 | Sox9 | Phf15 |
| Prickle2 | Pwwp2a | Zbtb39 | Dgke | Stac2 |
| Rhobtb1 | Ypel2 | Myb | Specc1 | Notum |
| Ptdss2 | Traf3 | Map3k5 | Pex12 | Jup |
| Tnik | Wdr35 | Gria1 | Dnmt3a | Stc2 |
| Sorbs2 | Six1 | Nlk | Jarid2 | Smoc1 |
| Ticrr | 4933426M11Rik | Phf15 | Pde4d | Irx2 |
| Mitf | Pcnx | Stac2 | Cdkal1 | Slc22a23 |
| Ppm1l | Dock4 | Jup | Tmem170b | Ror2 |
| Zfp319 | Itga2 | Tbc1d16 | Dip2c | Atxn1 |
| Tet2 | Anxa8 | Zfp354c | Mtx3 | Id4 |
| Ctsc | Samd8 | Stc2 | Zswim6 | Ogdhl |
| Hivep1 | Ptk2b | Sox11 | Ddx46 | Slc1a3 |

| | | | | |
|---|---|---|---|---|
| Fmn2 | Arhgef3 | Ltbp2 | Arl15 | Dennd3 |
| Cpne4 | Adamts12 | Pomt2 | Jmy | Npr3 |
| Fam167a | Vps13b | 1110018G07Rik | Slmap | Pou6f1 |
| Sema5a | Tmem117 | Fos | Gpc6 | Mgat3 |
| Tmem56 | Map4k3 | Slc22a23 | Adk | Fgd4 |
| Med13l | Abca3 | Ptch1 | Ptger4 | Cxadr |
| Vps13c | Zfp318 | Ror2 | Amigo2 | Clip4 |
| Fbln1 | Chd6 | Akr1c14 | 2410089E03Rik | Mpp7 |
| Diexf | Optn | Lyst | Card10 | Asxl3 |
| Slitrk6 | Gpr155 | Gli3 | Osmr | Cdc42bpg |
| Kcnq5 | Ncoa3 | Nid2 | Robo1 | Sufu |
| Pik3r4 | Phf20 | Fbxo34 | Robo2 | Afap1l2 |
| Vav3 | Tet2 | Atg14 | Bbx | Slco4a1 |
| Ccdc141 | Unc5c | Ogdhl | Arhgap28 | Rnf24 |
| Slc2a3 | Col11a1 | 5031414D18Rik | Prepl | Abca2 |
| Tbc1d4 | Ssbp3 | Abcc4 | Cyp39a1 | Prnd |
| Dscam | Ctnnbip1 | Slc1a3 | Heatr5b | Ldlrad3 |
| Depdc1b | Foxj3 | Lifr | Smoc2 | Sort1 |
| Mkx | Ldlrap1 | Dennd3 | Arid1b | Nup210l |
| Adra2a | Cachd1 | Npr3 | Pard6g | Tbc1d2 |
| Itpr1 | Nfxl1 | Fam84b | Zfp407 | Zfp618 |
| L3mbtl3 | Fryl | Vps8 | Cd14 | Prdm16 |
| Epha4 | 8430419L09Rik | Alcam | Tshz1 | Inadl |
| Itga11 | Zfp212 | D16Ertd472e | Cables1 | Chd7 |
| Adcyap1 | Gxylt2 | Hunk | Sorcs1 | Tbx3 |
| Id4 | Arrb1 | Dgkg | Tcf7l2 | Ttyh3 |
| Krba1 | Fgfr2 | Gnb1l | Rfx3 | Rundc3b |
| Pou2f1 | Foxf1 | Snx29 | Atrnl1 | Plxnd1 |
| Hectd3 | Lig4 | Pcyt1a | Rab11fip2 | Pparg |
| Zhx2 | Sfrp1 | Crkl | Jak2 | Bhlhe41 |

| | | | | |
|---|---|---|---|---|
| Pde1a | Rbp1 | Cxadr | Tmem2 | Dennd5b |
| Nbas | Myo6 | Parn | Gfra1 | Tspan11 |
| Whamm | Pik3r4 | Emilin2 | Btbd3 | Adam12 |
| Zfat | Arhgef12 | Cdkn1a | Kif5c | Whamm |
| Fbxw9 | Ube2cbp | Setbp1 | Rad51 | Pcsk6 |
| Itga2 | Nod1 | Slc26a2 | Cd93 | Dusp4 |
| Stard8 | Zbtb40 | Mpp7 | Fbn1 | Agpat5 |
| Afap1l2 | Daam1 | Elac1 | Rassf2 | Stac |
| Rgs17 | Cyp26b1 | Asxl3 | Secisbp2l | Tbx18 |
| Hlx | Slco3a1 | Gbf1 | Tbl1xr1 | Rasl12 |
| Nell2 | Prickle2 | Zfyve27 | Rap2b | Acpl2 |
| Zfp839 | Ptdss2 | Sufu | Dclk1 | Smad6 |
| E2f7 | Ppm1l | Hif1an | Fnbp1l | Nhej1 |
| Arap3 | Zfp319 | Lrrc4c | Ank2 | Lgr6 |
| Kctd1 | Hivep1 | Plcb1 | Syde2 | Il18rap |
| Ptprd | Cpne4 | Frmd5 | Syt11 | Sft2d2 |
| Fbn2 | Fam167a | Slco4a1 | Rab33b | Cnnm3 |
| ORF63 | Tmem56 | Rnf24 | Arnt | Fbxo28 |
| Slc39a8 | Med13l | Brd3 | Nbea | Diexf |
| Lrrc16a | Vps13c | Il1rn | Foxo1 | Rnpepl1 |
| Ern1 | Vav3 | Zfp120 | Runx1t1 | Ppp1r12b |
| Zc3h13 | Mkx | Ap4e1 | Kif1b | Vangl2 |
| Ttc7b | Adra2a | Abca2 | Epb4.1 | En1 |
| Cyth1 | Itga11 | Vps18 | Mdn1 | Stxbp5 |
| Rtl1 | Pde1a | Ehmt1 | Snx30 | Rhobtb1 |
| Fryl | Nbas | Ldlrad3 | Nfib | Mier2 |
| Pi15 | Rgs17 | Fat4 | Ncor2 | R3hdm2 |
| Abca3 | Ptprd | Pde5a | Bri3 | Chst3 |
| Bcas3 | Lrrc16a | Npnt | Tbc1d14 | Timeless |
| Flrt3 | Flrt3 | Gstm4 | Lrpap1 | Slc29a3 |

| | | | | |
|---|---|---|---|---|
| Rbm47 | Reep1 | Sort1 | Zfp326 | Sbno2 |
| Bhlhe22 | Ank3 | Nup210l | Akap9 | Foxo3 |
| Reep1 | Cenpj | Efna1 | Ube3b | Adra1b |
| Ankrd44 | Ikzf2 | Il6ra | A430033K04Rik | Med9 |
| Ncoa1 | Map3k1 | Zfp618 | Fam126a | Smtn |
| Pcnt | D430042O09Rik | Abca1 | Jhdm1d | Bahcc1 |
| Ank3 | Sik1 | Hsdl2 | Lrp6 | Mnt |
| Pcnx | Trim71 | Ttc39b | Plekha5 | Meox1 |
| Helz | Asap2 | Pik3cd | Ube2h | BC030867 |
| Scfd2 | Mgat5 | Lyn | Dusp16 | Plscr3 |
| Lama4 | Ssh2 | Alg2 | Atf7ip | Osbpl7 |
| Flt4 | Epha4 | Zkscan1 | Stk38l | Unk |
| Cenpj | Id4 | Rgs12 | Pdzrn3 | Slc6a4 |
| Zfp760 | Whamm | Rhbdd2 | Zfp507 | Fbf1 |
| Slc39a3 | Afap1l2 | Ttyh3 | Akap13 | Tmem199 |
| Nudt14 | Ankrd44 | Rundc3b | Zfp74 | Pdlim4 |
| Ikzf2 | Tbx3 | Fzd1 | Ate1 | Tom1l2 |
| Snx24 | Adam12 | Kcnh2 | Rsf1 | Mtfp1 |
| Slc15a2 | Notum | Phtf2 | Herc2 | Stk10 |
| Akap7 | Smoc1 | Trrap | Chd9 | Cacna1g |
| Sec16b | Chd7 | Zfp68 | Insr | Hid1 |
| Map3k1 | Fgd4 | Zfp605 | Lonrf1 | Cntrob |
| Ttc7 | Ralgps2 | Gm15800 | U2surp | Slc43a2 |
| Med9 | Pou6f1 | N4bp2 | Rab8b | E130012A19Rik |
| Fgfr2 | Dennd5b | Rbm47 | Hinfp | Map3k14 |
| Wars2 | Abr | Pcdh7 | Herc1 | Lif |
| Pgm2l1 | Mpp2 | Sspn | Col12a1 | Ppp1r13b |
| Slit3 | Lss | Plxnd1 | Dennd4a | Nudt14 |
| Gem | | Pparg | | Tnfaip2 |
| Rexo1 | | Bhlhe41 | | Six1 |

Tbx3
Tinagl1
Thnsl2
Mink1
Atp6v0a1
Tom1l2
Sipa1l3
Wdr35
Traf3ip1
Plekhm1
Adam12
Prkce
Itga8
Depdc5
Anks1
Kcnab1
Cradd
Ncald
Trerf1
Notum
Cachd1
Gm17296
Smoc1
Tmem87b
Lsm11
Adc
Exd2
D430042O09Rik
Lrch1
Zfp239

Avl9
Tspan11
Copg2
Ppm1k
Zfp84
Zfp790
Pcsk6
Dusp4
Agpat5
Tln2
Tbx18
Zfp445
Acpl2
Cdon
Ano10
Zfp609
Csnk1g1
Atp7a
Perp
Irx2
Atxn1
Mgat3
Clip4
Cdc42bpg
Prnd
Tbc1d2
Inadl
Prdm16
Stac
Rasl12

Zfyve21
Zfp36l1
Ahr
Foxq1
Edn1
Depdc1b
Itga2
Fam65b
Rnf44
Zmiz1
Egr3
Ptk2b
Lrch1
Tbc1d4
Cdh6
Cdh10
Pdgfb
Fam83h
Pick1
Lmbr1l
Wnt10b
Mkl1
Mapk11
Trmu
Tmem117
Itsn1
St6gal1
Scarf2
Nfkbiz
Runx2

Dusp8
Chd7
Pecr
Ptpn23
8430419L09Rik
Ahi1
Clasrp
Asb1
Dact3
Mical3
Fam188b
Fgd4
Mapre2
Gch1
Nup210l
Ralgps2
Fchsd2
Osbpl6
Rorc
Gpr173
Sik1
Msra
Trim71
Itgb6
Ppp3cc
Ppm1k
Myo6
Nudt16
Mtfp1
Optn

Smad6
Epha4
Id4
Whamm
Afap1l2
Ankrd44
Tbx3
Adam12
Notum
Smoc1
Chd7
Fgd4
Ralgps2
Pou6f1
Dennd5b
Abr
Mpp2
Lss

Cdc42ep3
Synj2
Mrps18b
Arap3
Pdgfrb
Dagla
Ccdc86
Mark2
Clcf1
Atg2a
Cd248
Ltbp3
Ubtd1
Kazald1
Neurl1a
Fas
Abtb2
Gpr155
Ggt7
Tmem189
Accs
Snapc4
Ambra1
Smox
Rbm38
Unc5c
Lef1
Wars2
4632415L05Rik
Cyr61

Pou6f1
Tecpr2
Phf15
Akr1c18
Mctp1
Cand2
Ldlrap1
Zfp583
Pdss2
Dennd5b
Arrb1
Cdkn1a
Mier2
Gpr155
Asap2
Rapgef3
Lhx2
Phf8
Esx1
Mgat5
Foxj2
Sec16a
N4bp2
Foxd1
Abr
Rasa4
Ube2cbp
Adamts12
Srl
Ttc28

Pear1
Muc1
Dclk2
Mtap
Ephb2
Tmem201
Col16a1
Col27a1
Coro2a
Spsb1
Pigo
Rusc2
Nbl1
Nsun4
Ldlrap1
Hectd3
Wdtc1
Dbc1
Sorcs2
Pom121
Sh2b2
Lfng
Usp42
Ung
Tchp
Ccdc92
Flt1
Scfd2
Cacna1c
Gata2

Megf10
Epg5
Lhpp
Bahcc1
Iqce
Ptpn13
Pom121
Abtb2
Ankrd13b
Mpp2
Ass1
Map3k5
Osbpl10
Atg9a
Gpr64
Dagla
Ophn1
Ino80
Lss
Dpf1
Panx1
Sh3bp2
Abcc4
Mertk
BC030867
Sesn2
Unk
St3gal3
Brsk1
Zfp821

Eif2ak3
Rab43
Foxj2
Ift122
C1s
Iqsec1
Gprin3
Clec2l
Gxylt2
Smpd1
Dact3
Megf8
Wnt11
Suv420h2
Spred3
Zfp36
Sipa1l3
Clasrp
Gdpd5
D8Ertd82e
Gm17296
Foxf1
4632415K11Rik
Acta1
Slc38a7
Mfhas1
Pnpla6
Lphn1
Banp
Taf1c

Ptprf
Cd200
Igfbp2
Csf1r
Arid5a
Snrk
P2ry2
D330045A20Rik
Cox10
Gnb1l
Atg2a
Dclk2
Bhlhe41
Kank1
Car12
Ccdc92
Egr2
Nhej1
Tnni3k
Dock4
Slc26a2
Ssh2
Pif1
Smyd4
Tchp
Nsun4
Itpkb
Micall2
Smcr7
Prokr1
Arhgef18
Ctsh
Tmem158
Lca5
Rbp1
Xylb
Acsbg1
Pde4a
Slc26a6

Sfxn2
Slc25a45
Rusc2
Ambra1
Rhobtb2
Abtb1
Lrp4
Cnnm3
Eepd1
E130308A19Rik
Cd53
Dcaf5
Dusp9
Prss22
Fadd
Ropn1l
Aars2
0610009L18Rik
Tnfaip2
Spon2
Bbs4
Dact1
4632415L05Rik
Ggt7
Slco4a1
Afap1
5730559C18Rik
Fzd5
Mbd4
Golgb1

Tnrc18
Lmtk2
Madd
Stim1
Atp13a2
Ick
Gdpd5
BC053749
Arhgap31
Bcl7a
Uaca
Vps18
Rps6ka5
Cep250
Nprl3
Cog7
Pknox2
Fchsd1
Ncoa2
Phf19
Slc17a6
Gmeb2
Slc4a3
Mtor
Pqlc2
Man2c1
Slco5a1
C2cd2l
Usp42
Wdr62

Pknox1
Anks6
Dnajc12
Ltbp2
Gm608
Mfhas1
Slc9a8
Abca2
Fzd1
Ptprm
Spry1
Nrp2
Cdon
Igf2bp2
Crybg3
Tbc1d22b
Med10
Gfra2
Mb21d2
Mylk
Apcdd1
Akna
Tmem194b
Nfatc1
Tns1
Adcy9
Bbs9
Lhx6
Scarf2
Sorbs3

Atg10
Ksr1
Lrp5
Cubn
Zfp521
Sardh
Prkdc
Bcl2l11
Sft2d2
Kitl
Fbxl8
Ebf1
Hook2
Rnf144b
Maml3
Socs1
Bcl6
Sox11
Eya1
Acsbg1
Cxadr
Rab15
Lpin1
Rgma
Calcrl
Tmem158
Pcsk6
Prex2
Adcy1
Tspan12

Pdgfra
Cpa6
Tlr2
Pitpnc1
St6gal1
2510009E07Rik
Ntn1
Slc22a23
Mef2c
Per2
Hunk
Setbp1
Dennd3
Lyn
Heg1
Hspa12b
Fam129a
Smox
